# Haploinsufficiency of two histone modifier genes on 6p22.3, *ATXN1* and *JARID2*, is associated with intellectual disability

**DOI:** 10.1186/1750-1172-8-3

**Published:** 2013-01-07

**Authors:** Tuva Barøy, Doriana Misceo, Petter Strømme, Asbjørg Stray-Pedersen, Asbjørn Holmgren, Olaug Kristin Rødningen, Anne Blomhoff, Johan Robert Helle, Alice Stormyr, Bjørn Tvedt, Madeleine Fannemel, Eirik Frengen

**Affiliations:** 1 Department of Medical Genetics, University of Oslo, P.O. Box 1036, Blindern, Oslo, N-0315, Norway; 2 Women and Children's Division, Department of Clinical Neurosciences for Children, Oslo University Hospital, Ullevål, Norway; 3 Faculty of Medicine, University of Oslo, Oslo, Norway

**Keywords:** 6p22-p24 deletion, 6p22.3, aCGH, ATXN1, Behavioural abnormalities, DTNBP1, Gait disturbance, Intellectual disability, JARID2

## Abstract

**Background:**

Nineteen patients with deletions in chromosome 6p22-p24 have been published so far. The syndromic phenotype is varied, and includes intellectual disability, behavioural abnormalities, dysmorphic features and structural organ defects. Heterogeneous deletion breakpoints and sizes (1–17 Mb) and overlapping phenotypes have made the identification of the disease causing genes challenging. We suggest *JARID2* and *ATXN1,* both harbored in 6p22.3, as disease causing genes.

**Methods and results:**

We describe five unrelated patients with *de novo* deletions (0.1-4.8 Mb in size) in chromosome 6p22.3-p24.1 detected by aCGH in a cohort of approximately 3600 patients ascertained for neurodevelopmental disorders. Two patients (Patients 4 and 5) carried non-overlapping deletions that were encompassed by the deletions of the remaining three patients (Patients 1–3), indicating the existence of two distinct dosage sensitive genes responsible for impaired cognitive function in 6p22.3 deletion-patients. The smallest region of overlap (SRO I) in Patients 1–4 (189 kb) included the genes *JARID2* and *DTNBP1*, while SRO II in Patients 1–3 and 5 (116 kb) contained *GMPR* and *ATXN1.* Patients with deletion of SRO I manifested variable degrees of cognitive impairment, gait disturbance and distinct, similar facial dysmorphic features (prominent supraorbital ridges, deep set eyes, dark infraorbital circles and midface hypoplasia) that might be ascribed to the haploinsufficiency of *JARID2.* Patients with deletion of SRO II showed intellectual disability and behavioural abnormalities, likely to be caused by the deletion of *ATXN1*. Patients 1–3 presented with lower cognitive function than Patients 4 and 5, possibly due to the concomitant haploinsufficiency of both *ATXN1* and *JARID2*. The chromatin modifier genes *ATXN1* and *JARID2* are likely candidates contributing to the clinical phenotype in 6p22-p24 deletion-patients. Both genes exert their effect on the Notch signalling pathway, which plays an important role in several developmental processes.

**Conclusions:**

Patients carrying *JARID2* deletion manifested with cognitive impairment, gait disturbance and a characteristic facial appearance, whereas patients with deletion of *ATXN1* seemed to be characterized by intellectual disability and behavioural abnormalities. Due to the characteristic facial appearance, *JARID2* haploinsufficiency might represent a clinically recognizable neurodevelopmental syndrome.

## Background

Deletions involving chromosome 6p22-p24 have previously been reported in 19 patients (18 patients reviewed by Celestino-Soper [1] and one described in [2]). In addition, an electively aborted 27 week-old fetus with a *de novo* deletion in 6p22.3-p24.3 was described with multiple malformations [3]. Most reported subjects were developmentally delayed and presented with a heterogeneous pattern of dysmorphic facial features, including various eye abnormalities, and congenital anomalies, including craniofacial malformations, kidney- and heart defects. Different breakpoints and sizes (1 to 17 Mb) of the deletions might account for a large part of this variability. However, based on the deletion overlap in their patient and six out of seven previously reported subjects, Bremer *et al.* proposed that the critical gene(s) were located in 6p22.3 [4].

We describe five unrelated patients with variable degree of cognitive impairment ranging from borderline IQ to severe intellectual disability, and *de novo* deletions (0.1-4.8 Mb) within 6p22.3-p24.1 detected by aCGH analysis. Two non-overlapping deletions in Patients 4 and 5 defined the two smallest regions of overlap (SRO I and II, 189 and 116 kb, respectively) in our patients, each deleted in four of the five patients. Both regions were located in 6p22.3 and contained only two genes each, thereby facilitating the identification of the disease causing genes in this region.

Based on their function as chromatin modifier genes that play a role in the Notch signaling pathway, we suggest that *JARID2* and *ATXN1* are likely to be the critical genes for cognitive function within SRO I and II, respectively. In addition, *JARID2* haploinsufficiency is likely to be the main contributor to the neurodevelopmental syndrome in Patients 1–4 consisting of impaired cognitive function, gait disturbance and distinct, similar dysmorphic facial features characterized by prominent supraorbital ridges, deep set eyes, dark infraorbital circles and midface hypoplasia.

## Methods

### Cytogenetic analysis

Chromosome metaphase spreads from peripheral blood of the five patients were analysed by standard G-banding methods.

### Array-comparative genome hybridization (aCGH)

DNA from leucocytes from the five patients and their parents was analysed by aCGH using one of the commercially available Human Genome CGH Microarrays 4x44K, 2x105A, 4x180K or 244A (Agilent Technologies Inc., Santa Clara, CA) according to the manufacturer’s recommendations. Samples were sex-matched with Human Genomic DNA (Promega, Madison, WI). Data were processed with Feature Extraction and DNA Analytics v4.0.76 (Agilent Technologies Inc.). All genomic positions were based on the February 2009 human reference sequence (GRCh37/hg19) by the Genome Reference Consortium.

### Real-time PCR

Quantitative real time-PCR (qPCR) amplifications were carried out on genomic DNA. Reverse transcriptase real time-PCR (RT-PCR) was performed on RNA from peripheral blood, after conversion of RNA to cDNA (High Capacity cDNA Reverse Transcription Kit, Life Technologies Corporation, Carlsbad, CA). SYBR Green JumpStart Taq ReadyMix (Sigma-Aldrich, Saint Louis, MO) chemistry was used for both qPCR and RT-PCR. Reactions were run in triplicate on the Applied Biosystems Real Time PCR 7900 HT Sequence Detector System according to the manufacturer’s recommendations. qPCR primers were designed at frodo.wi.mit.edu/primer3 [5], and the amplification levels were calculated using the 2^-ΔΔCt^ method [6]. RT-PCR primers targeting *JARID2* [GenBank: NM_004973.3] and *ATXN1* [GenBank: NM_001128164.1] transcripts were designed at ncbi.nlm.nih.gov/tools/primer-blast (primer sequences supplied in Additional file 1: Table S1), and the amplification levels were calculated according to Vandesompele *et al.*[7]. Dissociation curve analysis revealed a single product for each primer pair.

### Growth standard curves

Norwegian consensus anthropometric measures were used in this report [8].

### Clinical reports

**Figure 1:**
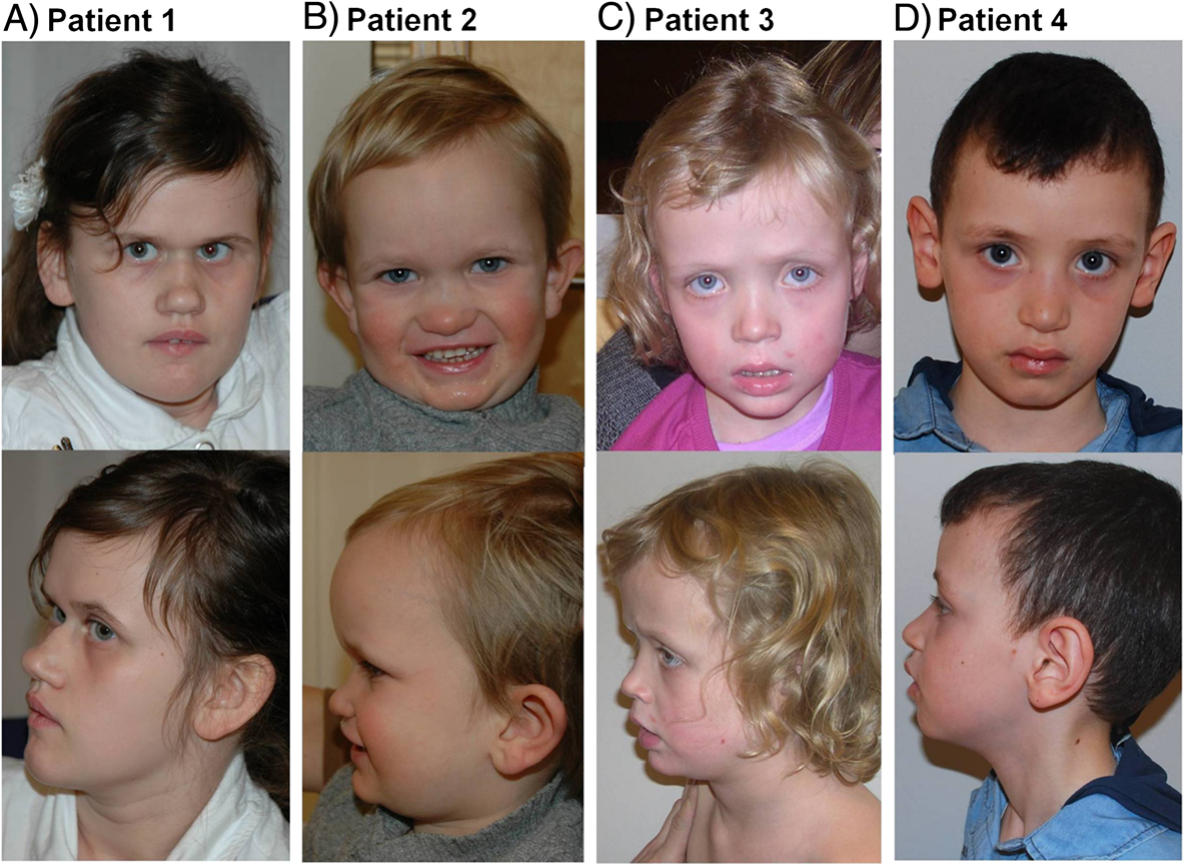
**Facial appearance of Patient 1 at the age of 13 years (A), Patient 2 at the age of 4 years (B), Patient 3 at the age of 6.5 years (C), and Patient 4 at the age of 5.5 years (D).** All patients have distinct, similar dysmorphic facial features, including prominent supraorbital ridges, deep set eyes, dark infraorbital circles and midface hypoplasia.

Patient 1 (reported in DECIPHER database with identification # 256835) was a 15-year-old girl, the second child to non-consanguineous healthy Norwegian parents. Pregnancy and delivery were uneventful, with the following birth measures: weight 4470 g (97^th^ centile), length 55 cm (>97^th^ centile) and occipitofrontal circumference (OFC) 36 cm (75^th^ centile). Global developmental delay was present from early childhood and she walked independently at 3.5 years of age. Testing with Reynell Developmental Language Scales and British Picture Vocabulary Scale at age 9 years, and Leiter International Performance Scale-Revised (Leiter-R) at age 9 and 10 years, indicated a mental age between 3 and 4 years, with expressive language below 3 years. The test results were consistent with moderate intellectual disability (IQ 35–49). A cerebral magnetic resonance imaging (MRI) examination at age 8 years showed unspecific periventricular white matter changes. She has had seizure-suspected syncope episodes from 13 years of age, but an electroencephalogram (EEG) was normal. At the last examination, at age 15 years, her weight was 53.7 kg (50^th^ centile), height 157.5 cm (10^th^ centile) and OFC 54 cm (25^th^ centile). Her vocabulary consisted of 20–30 words, no sentences, and her articulation was blurred. She was hyperactive with a short concentration span, and had sleeping difficulties. She demonstrated unsteady and broad-based gait with difficulties standing on one leg at a time, and a remarkable inability to jump on one as well as on two legs (Additional file 2: Video Patient 1). Dyspraxia was suggested by a discrepancy in performance between on-command and self-initiated tasks and language. Muscle tone was normal. Hearing and vision were normal. Ultrasound scan of kidneys was normal. She had pes planovalgus, short halluxes and apparently long 2^nd^ toes bilaterally. X-ray images of hands and feet revealed shortening of the 1^st^ and 3^rd^ -5^th^ metacarpal bones of the left hand and the 1^st^ and 5^th^ metacarpal bones of the right hand, and bilateral short 1^st^ metatarsal bones (Additional file 3: Figure S1). Dysmorphic facial features (Figure 1A) and minor congenital anomalies in this patient are listed in Table 1.

**Table 1:** Summary of the aCGH results and clinical data of presently reported patients with 6p22.3-p24.1 deletions

**Patient**	**1**	**2**	**3**	**4**	**5**
Gender (M/F)	F	M	F	M	F
Age at last follow-up (y)	15	4	6.5	6.5	17
**Deletion**					
Chromosomal band	6p22.3-p24.1	6p22.3-p23	6p22.3-p23	6p22.3	6p22.3
Position (bp, hg19)	13071924-17918584	15081747-18207178	14545576-16846846	15405377-15594945	16215803-16332297
Deleted candidate genes	JARID2, ATXN1	JARID2, ATXN1	JARID2, ATXN1	JARID2	ATXN1
Size (kb)	4846	3125	2301	189	116
Agilent array	105A	180K	44K	105A	244A
**Face**					
Broad forehead	+	+	+	-	-
Prominent forehead	-	+	-	-	-
Frontal bossing	+	-	+	-	-
Prominent supraorbital ridges	+	+	+	+	-
Deep set eyes	+	+	+	+	-
Hypotelorism	+	-	-	-	-
Hypertelorism	-	-	+	-	-
Strabismus	+	-	-	+	-
Infraorbital dark circles	+	+	+	+	-
Midface hypoplasia	+	+	+	+	-
Deep set nasal root	+	+	-	+	-
Bulbous nasal tip	+	+	+	+	-
Short philtrum	+	+	-	+	-
Full lips	+	+	+	+	-
Marked Cupid's bow	+	+	+	+	-
Accentuated central depression lower lip	+	+	-	+	-
Prominent or pointed chin	+	+	+	+	-
**Neurology**					
Intellectual disability	Moderate	Moderate	Severe	Borderline	Mild
Gait disturbance	+	+	+	+	-
Dyspraxia	+	?	?	+	-
Muscle tone	Nl	Nl	Nl (neonatal hypotonia)	Nl (early-onset hypotonia)	Nl
Seizures	+	+	-	-	-
Behavioural abnormalities	Hyperactivity, sleeping difficulties	Poor eye contact, repetetive behaviour	ASDs, hyperactivity, sleeping difficulties	Nl	Psycotic episode, aggression, hyperactivity, repetetive behaviour
**Other**					
Structual heart defects	NE	Atrial septal defect	-	NE	NE
Umbilical hernia	-	-	+	-	-
Kyphosis	+	-	-	-	-
Hand anomalies	Cl, variable short metacarpal bones	Cl	Cl	Cl	-
Foot anomalies	Pes planovalgus, Sy 2–4 toes, SG, crooked 4th toes ul 3rd toes, short and broad H, variable short metatarsal bones	Pes planovalgus, Sy 2–4 toes	Ul 5th toes, 2nd and 4th toes lying over 3rd toes	Sy 2–3 toes, SG, broad H	-
Dental anomalies	Pointed dens caninus	-	Irregularly placed frontal teeth	-	-

Legend: +, feature present; -, feature absent; ?, not cooperative for sufficient assessment, ASDs; autism spectrum disorders; Cl, clinodactyly 5th fingers; F, female; H, halluxes; M, male; NE, not examined; Nl, normal; SG, sandal gap; Sy, syndactyly; Ul, underlying.

Patient 2 was a 4-year-old boy, the third child to non-consanguineous healthy Norwegian and English parents. Pregnancy and delivery were uneventful, with the following birth measures: weight 4090 g, length 52 cm and OFC 36 cm (all measures at the 75^th^ centile). From an early stage his eye contact and speech were delayed, and he walked independently at 2 years of age. Testing with Bayley Scales of Infant and Toddler Development, third edition (Bayley III) at age 3.5 years showed cognitive functioning corresponding to 1.5 years, suggesting moderate intellectual disability. He did not fulfill the criteria for autism spectrum disorders (ASDs) according to the Autism Diagnostic Observation Schedule (ADOS). At the same age, a cerebral MRI examination was normal. Seizure-suspected syncope episodes occurred during infancy. EEG performed at age 8 months was normal, whereas cardiologic work-up revealed an atrial septal defect that closed spontaneously. At the last examination, at age 4 years, his weight was 19 kg (90^th^ centile), height 105 cm (75^th^ centile) and OFC 52 cm (50^th^ centile). His language development was severely delayed; he could only speak a few words. He had a broad-based and unsteady gait (Additional file 4: Video Patient 2) and seemed unable to stand on one leg at a time. Muscle tone was normal. Hearing and vision were normal. Dysmorphic facial features (Figure 1B) and minor congenital anomalies in this patient are listed in Table 1.

Patient 3 was a 6.5-year-old girl, the first of two children to non-consanguineous healthy Norwegian parents. Pregnancy and delivery were uneventful, with the following birth measures: weight 3470 g, length 49 cm and OFC 34 cm (all measures at the 25^th^ centile). A large umbilical hernia was noted at birth. She had muscular hypotonia that persisted for 3 to 4 months. She crawled at 12 months and walked at 18 months. At 5 years and 10 months testing with Bailey II, she showed cognitive functions corresponding to 12 to 13 months, suggesting severe intellectual disability (IQ 20–34). Evaluation with ADOS showed behaviour consistent with ASDs. A cerebral MRI examination at 3 months was normal. In the neonatal period she had a few episodes of apnea with cyanosis of unknown cause, but EEG was normal except for unspecified slow wave activity, and cardiologic work-up was normal. At the last examination, at age 6.5 years, her weight was 24.7 kg (50^th^ centile), height 127 cm (90^th^ centile) and OFC 53.5 cm (75-90^th^ centile). Her language development was severely delayed with a vocabulary of less than 50 words. She could not speak in sentences. She was hyperactive with an unsteady gait (Additional file 5: Video Patient 3), had sleeping difficulties and still used diapers. Muscle tone was normal. Hearing and vision were normal. Dysmorphic facial features (Figure 1C) and minor congenital anomalies in this patient are listed in Table 1.

Patient 4 was a 6.5-year-old boy, the second of four children to non-consanguineous Lebanese parents. The mother and maternal uncle had epilepsy. Pregnancy and delivery were uneventful, with the following birth measures: weight 3756 g, length 51 cm and OFC 35 cm (all measures at the 50^th^ centile). He walked at age 16 months. When tested at age 4 years and 3 months with Reynell Developmental Language Scales, his language development was delayed by 1.5 years. Testing with WPPSI-III at age 6.5 years gave a borderline IQ of 74 (borderline intellectual functioning: IQ 70–84). He performed best on verbal tests, despite being bilingual. Cerebral MRI and EEG examinations at age 6 years were normal. At the last examination, at age 6.5 years, his weight was 23.9 kg (50^th^ centile), height 126 cm (75^th^ centile) and OFC 52 cm (25^th^ centile). He had balance and coordination problems (Additional file 6: Video Patient 4). Finger opposition was inadequate and he performed poorly when asked to stand on one leg at a time, jump, or walk on his toes or heals. Muscle tone was normal. Hearing and vision were normal. Due to early-onset muscular hypotonia he had been extensively examined for the possibility of neuromuscular disease, however, with normal results. Testing for metabolic abnormalities was also normal. Dysmorphic facial features (Figure 1D) and minor congenital anomalies in this patient are listed in Table 1.

Patient 5 was a 17-year-old girl, born as the second of three children to non-consanguineous Norwegian parents. The mother had Sjogren syndrome. The pregnancy was complicated by episodes of maternal bleeding. Delivery was normal, with the following birth measures: weight 3800 g, length 52 cm (both at the 75^th^ centile) and OFC unknown. She walked independently at 14 months, and developed the stereotypic habit of constantly walking around. At 3 years and 3 months she showed delayed language and motor development, and hyperactivity. Testing with Bayley-II gave a Mental Developmental Index of 55, corresponding to a developmental age of approximately 2 years. Her language development was particularly delayed, and she lacked concentration and impulse control. At 8 years WISC-R gave an IQ of 60. A recent testing with WISC-IV gave an IQ slightly below 70, and Wechsler Adult Intelligence Scale-Fourth Edition (WAIS-IV) and other tests were in accordance with this, indicating mild intellectual disability (IQ 50–69). She attended a school for children with learning disabilities. A cerebral MRI examination at 6 years was normal. She has had episodes of uncontrolled anger outbursts, sometimes with violent behaviour, and at age 14 she had a psychotic episode. Over the years she has displayed stereotypic behaviour such as chewing on her fingers, but she has not been considered as having ASDs. At the last examination, at age 17 years, she was obese with a Body Mass Index (BMI) of 30.6 and height 162.5 cm (25^th^ centile). She had a mild unsteadiness when standing with her eyes closed. Muscle tone was normal. Hearing and vision were normal. Testing for fragile X syndrome and work up for detection of abnormal metabolites in the urine were normal. Dysmorphic features were not noted (consent to publish photos of this patient was not obtained).

## Results

**Figure 2:**
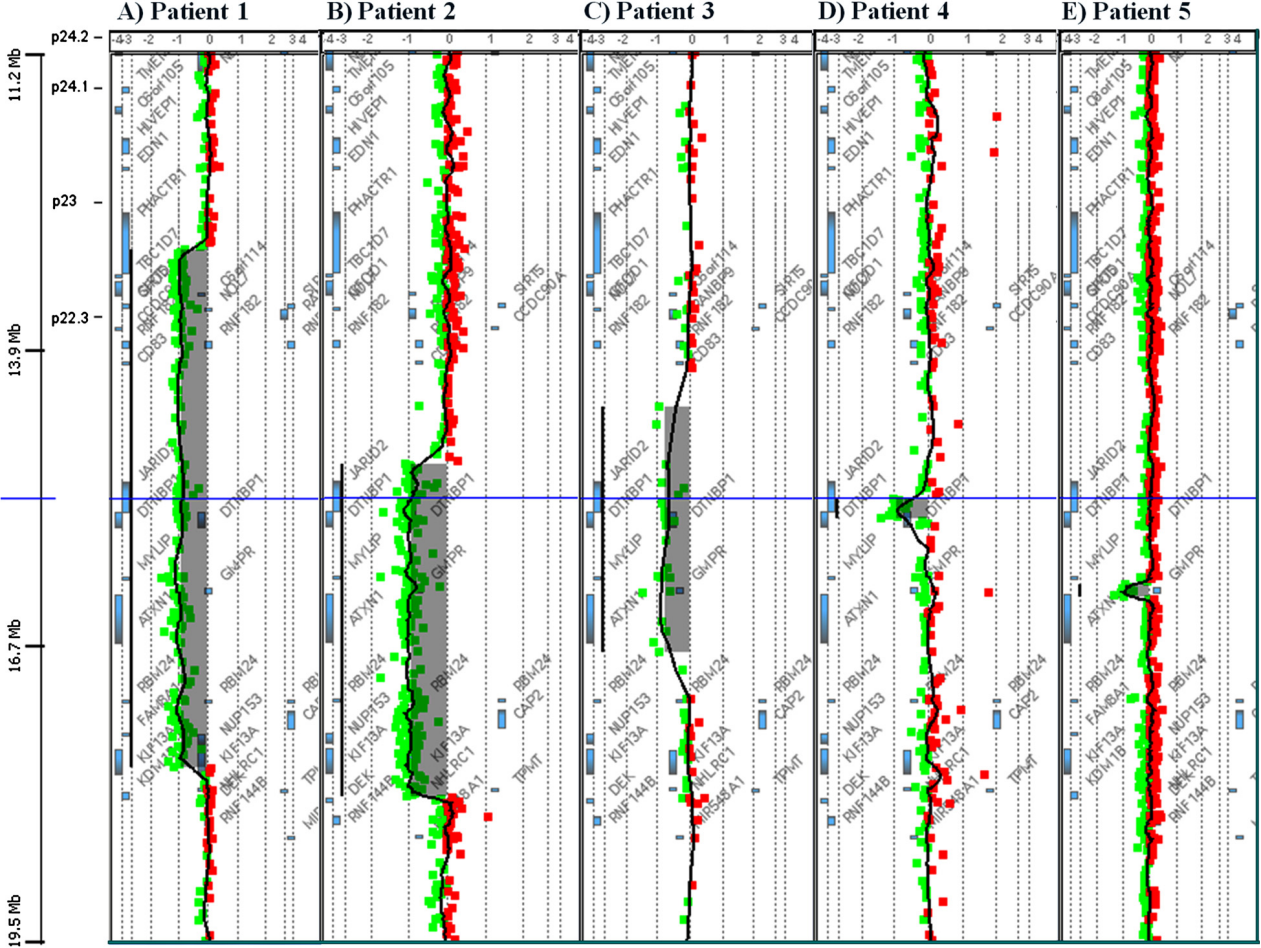
**Array-CGH results in Patients 1-5 (A-E) revealing deletions in chromosome 6p22.3-p24.1.** The shaded area indicates the deleted area with an average log2-ratio of -1, indicating loss of one copy of the genomic segment.

**Figure 3:**
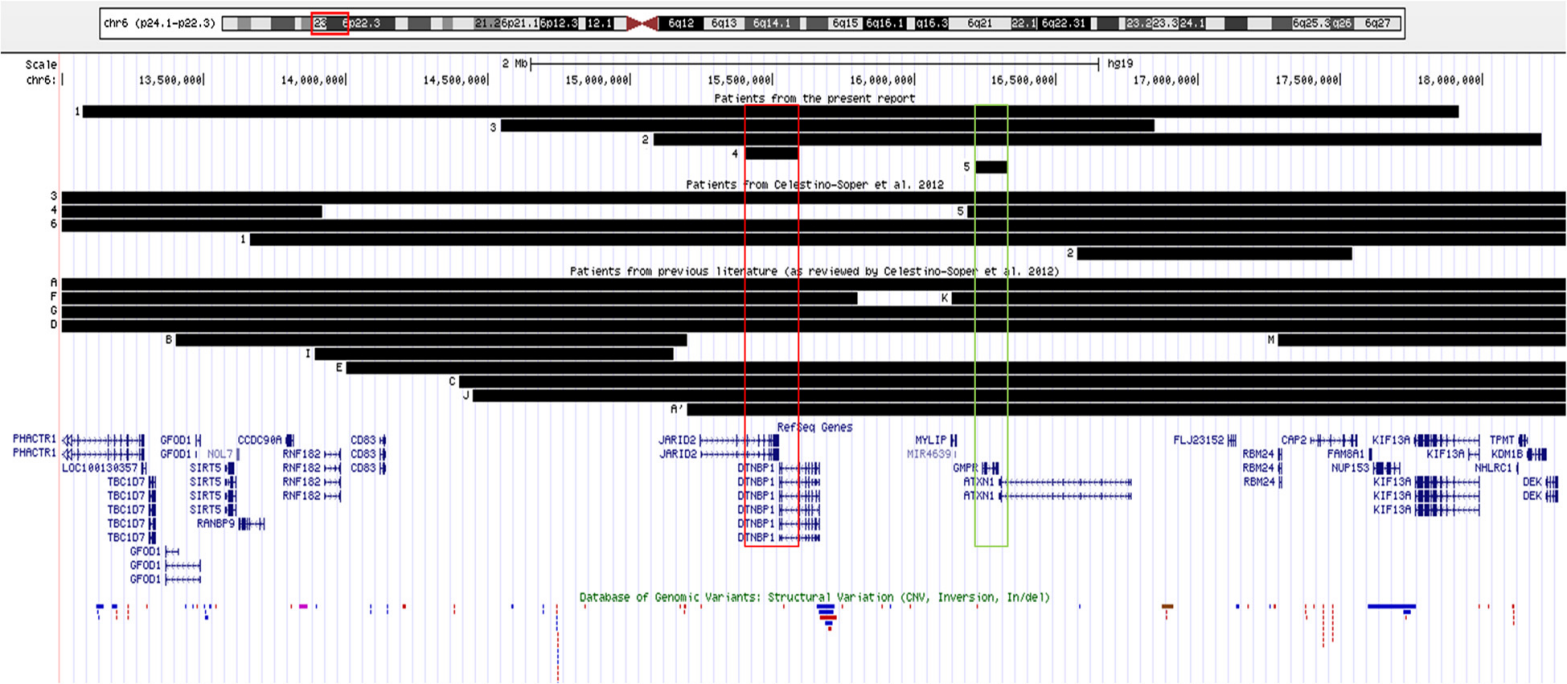
**Localization of the deletions in chromosome 6p22.3-p24.1 in our five patients (top) and the overlapping part of the deletions in 17 out of 19 previously reported patients.** SRO I in Patients 1-4 from the present report is 189 kb (red box), and SRO II in Patients 1-3 and 5 is 116 kb (green box). JARID2 and ATXN1 are deleted in 14 and 16 patients, respectively. Patient codes and deletion coordinates for previous patients are according to [1], except M from [2]. CNVs recorded in the Database of Genomic Variants (projects.tcag.ca/variation) (below) are covering only a minor part of the region deleted in the five patients. For the two genes located within the SRO I, a single CNV in JARID2 was located in an intronic sequence, and two CNVs were reported in DTNBP1: one located in an intronic sequence and one copy number gain including the 5’UTR and the first exon. For the two genes located within SRO II, no CNVs were reported in GMPR and one CNV, a gain in an intronic sequence observed in three individuals, was recorded in ATXN1. Data were uploaded into UCSC Genome Browser ( http://genome.ucsc.edu).

Karyotype analysis of all five patients was normal. Deletions in chromosome 6p22.3-p24.1 of 116 kb to 4.8 Mb in size were detected by aCGH analysis in the five patients (Table 1 and Figure 2 and 3). Patients 4 and 5 carried non-overlapping deletions that were both included in the deletions of Patients 1–3. Patient 1 had a deletion involving 6p22.3-p24.1 of 4.8-4.9 Mb and Patient 2 had a 3.1 Mb deletion overlapping the proximal 2.8 Mb of the deletion in Patient 1. Array analysis in Patient 3 revealed a 2.3-2.6 Mb deletion included in the region deleted in Patients 1 and 2, and the common region deleted in these three patients contained six genes: *JARID2, DTNBP1, MYLIP, MIR4639, GMPR* and *ATXN1.* In Patient 4, we identified a 189–241 kb deletion, including part of the genes *JARID2* and *DTNBP1*, which was the region defining SRO I, deleted in Patients 1–4. A 116–163 kb deletion was detected in Patient 5 including the gene *GMPR* and part of the gene *ATXN1*. This was the region defining SRO II, deleted in Patients 1–3 and 5. The partial deletion of *JARID2* and *DTNBP1* in Patient 4, and *ATXN1* in Patient 5 removed the 3’end of the genes, deleting exons 3–18 in *JARID2*, exons 7–10 in *DTNBP1* and exons 8–9 in *ATXN1* and the 3’ untranslated region (UTR) in all cases. Only copy number variants (CNVs) covering minor parts of the region deleted in our patients were recorded in the Database of Genomic Variants (DGV, projects.tcag.ca/variation, accessed in November 2012) (Figure 3). From the aCGH result it was uncertain if the gene *KDM1B* was deleted in Patient 2, as it was partly included in the region between the last deleted and the first normal aCGH oligo at the proximal border, and it was shown by qPCR not to be deleted. No additional genes were located in the region between the minimal and maximal sizes of the deletions, and the breakpoints of the deletions given by the aCGH results were therefore not investigated further. All five chromosome 6 deletions were verified, by qPCR (Patients 1–4) or by performing a second aCGH with higher resolution (Patient 5), and were found to be *de novo* by aCGH analysis of the parental samples. In addition, the aCGH analysis detected a 463 kb duplication of chromosome 4q23 in Patient 3 (chr4:100124832–100588141, bp) and a 94 kb deletion of chromosome 11p13 in Patient 5 (chr11: 32697424–32791452, bp). Two DECIPHER patients (identification # 250971 and # 256563) were reported with a duplication that overlaps the duplicated region in Patient 3. However, both duplications were larger (both about 3.6 Mb), and one of the individuals also had two other aberrations. Similarly, one DECIPHER patient (identification # 253354) had a deletion covering the region deleted in Patient 5, which also was larger (4.1 Mb). A minor part of the duplication of chromosome 4q23, and most of the deletion of chromosome 11p13 overlapped with CNVs in DGV. These aberrations in Patient 3 and 5 were inherited from healthy parents and the gene content did not appear relevant for the clinical presentation of the patients.

**Figure 4:**
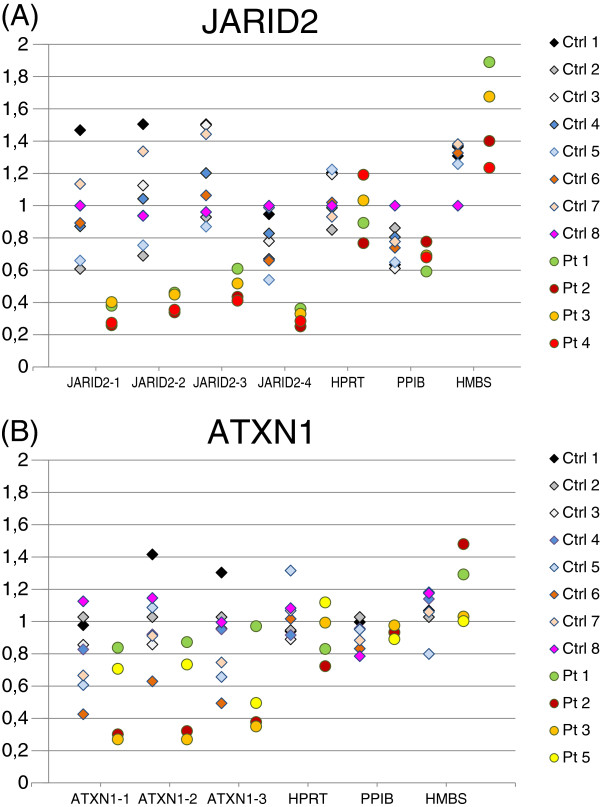
**Expression levels of JARID2 (A) and ATXN1 (B) in leucocytes from our four patients with deletion of one or both of these genes (Patients 1-4 and Patients 1-3 and 5, respectively), compared with eight healthy controls.** JARID2 and ATXN1 levels were measured using four and three primer pairs targeting the transcripts, respectively (JARID2-1, -2, -3 and -4, and ATXN1-1, -2 and -3). Primer sequences are given in Additional file 1: Table S1). JARID2 expression level was significantly reduced in the patients compared to the controls (p ≤ 0.01), while the ATXN1 expression level was not significantly altered (p ≤ 0.1). Ctrl, Control; Pt, Patient.

Expression of *JARID2* and *ATXN1* measured by RT-PCR was analyzed in the four patients that carried a deletion involving the gene(s) (Patients 1–4 and Patients 1–3 and 5, respectively) and in eight healthy controls, using RNA from leucocytes. The expression levels of *JARID2* and *ATXN1* were normalized to the levels of the three housekeeping genes, *HPRT*, *PPIB* and *HMBS*, which showed the most homogenous expression in the five patients among totally ten control genes analyzed. *JARID2* expression was significantly reduced in our patients compared to controls (p ≤ 0.01) (Figure 4A). *ATXN1* expression in the patients was not significantly different from controls (p ≤ 0.1), but a trend towards reduced expression was observed (Patients 2 and 3 had a reduced expression level) (Figure 4B).

## Discussion

Although deletions in 6p22-p24 have been suggested to be relatively rare, 19 patients have so far been studied [1,2,4,9-15]. We detected five new patients with *de novo* deletions in chromosome 6p22.3-p24.1 in a cohort of about 3600 patients investigated in our diagnostic laboratory on the indication developmental delay, ASDs and/or congenital abnormalities (Table 1 and Figure 2 and 3). The molecular and clinical data of all 24 patients are summarized in Table 2. Chromosome 6p22-p24 haploinsufficiency results in a clinically heterogeneous neurodevelopmental syndrome. However, most of the patients manifest developmental delay and variable degree of cognitive impairment, and various dysmorphic facial features. Neurological abnormalities, such as epileptic seizures, lack of coordination and/or muscular hypotonia were recorded in 16 patients. ASDs and/or behavioural abnormalities, including stereotypic behaviour, aggressiveness, attention deficits and motor hyperactivity were described in 12 patients. Nine patients had a structural heart defect, and eight patients had skeletal anomalies. The different breakpoints and sizes (0.1-17 Mb) of the deletions are likely to account for a large part of the phenotypical variability.

**Table 2:** Summary of deletion data and clinical presentation of all published patients with 6p22-p24 deletions

**Pt (a)**	**Reference**	**Gender**	**Age (b)**	**Chr6 coordinates (hg19) (c)**	**Size (Mb) (d)**	**DD/ID**	**Neurological abnormalities**	**ASDs**	**Behavioural abnormalities (e)**	**Structural heart defects**	**Skeletal anomalies**	**Dental anomalies**	**Hernia**	**Dysmorphic features**
1	Present report	F	15y	13071924-17918584 bp	4.8	+	+	-	+	N/A	+	+	-	+
2	Present report	M	4y	15081747-18207178 bp	3.1	+	+	-	+	+	N/A	-	-	+
3	Present report	F	6.5y	14545576-16846846 bp	2.3	+	+	+	+	-	N/A	+	+	+
4	Present report	M	6.5y	15405377-15594945 bp	0.2	+ (f)	+	-	-	N/A	N/A	-	-	+
5	Present report	F	17y	16215803-16332297 bp	0.1	+	(+) (g)	-	+	N/A	N/A	-	-	-
1	[1]	M	15y	13662096-19042218 bp	5.1	+	+	+	+	-	+	N/A	N/A	+
2	[1]	M	4y	16572367-17543199 bp	1.0	+	N/A	(+) (h)	+	N/A	N/A	N/A	N/A	-
3	[1]	F	1m	9621501-24218259 bp	14.6	N/A	N/A	N/A	N/A	+	N/A	N/A	N/A	+
4	[1]	M	17y	10269968-13915223 bp	3.6	+	+	-	+	-	+	N/A	N/A	+
5	[1]	F	7y	16186391-21421705 bp	5.2	+	+	-	N/A	N/A	N/A	N/A	N/A	-
6	[1]	M	3y	12058814-20896726 bp	8.8	+	+	-	+	+	+	+	N/A	+
A	[14]	M	3y	(2.3-4.2) – (25.2-27.0) or (13.4-15.2) – (30.4-32.1) Mb	N/A	+	+	N/A	N/A	+	+	N/A	+	+
B	[12]	M	9m	(7.1-13.4) – (15.2-25.2) Mb	N/A	N/A	+	N/A	N/A	+	N/A	N/A	+	+
C	[9, Pt 1]	M	15y	14.4 – 21.6 Mb	N/A	+	+	N/A	(+) (i)	N/A	N/A	+	+	+
D	[9, Pt 2]	F	13m	11.9 – 18.7 Mb	N/A	+	+	N/A	N/A	+	N/A	N/A	N/A	+
E	[10, Pt 91–145]	F	34m	(13.0-14.0) – 21.7 Mb	N/A	+	+	N/A	N/A	N/A	N/A	N/A	-	+
F	[10, Pt 95–800]	M	20y	10.0 – 15.8 Mb	N/A	+	+	N/A	+	N/A	+		-	+
G	[10, Pt PF]	M	4y	10.0 – 18.7 Mb	N/A	+	N/A	N/A	N/A	N/A	-	N/A	+	+
H	[11]	M	23m	(4.2-6.1) – (10.4-11.9) Mb	N/A	N/A	N/A	N/A	N/A	+	+	N/A	N/A	+
I	[13, Pt AU010604]	M	N/A	13889301-15153952 bp	1.3	N/A	N/A	+	N/A	N/A	N/A	N/A	N/A	N/A
J	[15]	F	16y	14446670-27741682 bp	13.3	+	+	N/A	+	+	+	+	N/A	+
K	[4]	F	4y	16132021-23152021 bp	7.0	+	N/A	-	-	+	N/A	N/A	-	+
L	[1]	N/A	N/A	18829825-23576125 bp	4.7	+	+	+	N/A	N/A	N/A	N/A	N/A	+
M	[2]	F	11y	17281809-24786325 bp	7.5	+	N/A	N/A	N/A	N/A	N/A	N/A	N/A	+
Total						20	16 (17)	4 (5)	10 (11)	9	8	5	5	20

Legend: +, feature present; ASDs, autism spectrum disorders; chr, chromosome; DD, developmental delay; F, female; ID, intellectual disability; M, male; m, month-old; N/A, information not available; Pt, patient; y, year-old; (a) codes for previous patients are according to [1], except from M [2]; (b) age at last examination; (c) deleted region for previous Patients 1–6 and A-L from [1], and in M deduced from the deleted genes noted by the authors [2]; (d) minimum size; (e) includes a wide spectrum of behavioural abnormalities, possibly also undiagnosed ASD; (f) borderline intellectual functioning (IQ =74); (g) possibly present; (h) suspected to have ASDs, but not formally tested; (i) poor concentration.

We detected the two smallest deletions within 6p22-p24 reported so far, defining two SROs in our five patients: SRO I in Patients 1–4 and SRO II in Patients 1–3 and 5, both located in 6p22.3 (Figure 3). Manifestation of cognitive impairment in both Patients 4 and 5, in spite of non-overlapping deletions, suggests that two dosage sensitive genes, within SRO I and II, independently contribute to cognitive impairment when deleted. Each SRO contained only two genes, allowing us to focus on candidate genes for the features shared by the patients.

In spite of the heterogeneous phenotype associated with 6p22-p24 deletions, we recognized a similar phenotype in our four patients with deletion of SRO I, consisting of gait disturbance and recognizable facial dysmorphic features, in addition to borderline IQ or intellectual disability. SRO I was defined by the 189 kb deletion in Patient 4, and included the genes *DTNBP1* and *JARID2*.

*DTNBP1*, dystrobrevin binding protein 1, encodes for dysbindin which is part of the Biogenesis of Lysosome-related Organelles Complex-1 (BLOC-1) [16]. BLOC-1 regulates intracellular protein trafficking and is implicated in the biogenesis of specialized organelles of the endosomal–lysosomal system [17]. *DTNBP1* genetic variants have been linked to general cognitive ability [18-20]. Furthermore, SNPs in this gene have been extensively associated with schizophrenia [21-23]. *In vitro* studies indicated dysbindin as a binding partner of several proteins with a suggested role in muscular physiology [24-26], and increased dysbindin transcript and protein levels were measured in muscle biopsies from individuals and mice with Duchenne Muscular Dystrophy (DMD; MIM 310200) [27,28]. However, muscle pathology was not reported in mutant *Dtnbp1* mice [16], and not detected in BLOC-1-deficient mice [29]. One patient with homozygous nonsense mutation in *DTNBP1* causing the recessive pigmentation and bleeding disorder Hermansky Pudlak Syndrome 7 (HPS7; MIM 614076) [16] was reported. Manifestations of cognitive impairment, muscle weakness, impaired balance and coordination, gait disturbance or dysmorphic features were not reported in this patient. Thus a link between *DTNBP1* haploinsufficiency and the features seen in our patients is unlikely.

The second gene included in SRO I, *JARID2* (jumonji, AT rich interactive domain 2), encodes a transcriptional repressor protein. Studies of the murine orthologous Jarid2 have shown that the encoded protein acts via methylation modifications that regulate developmental processes (reviewed by Takeuchi [30]), as well as organ homeostasis [31]. In particular Jarid2 has an important function in Prc2 and Notch1 pathways. Polycomb group (PcG) of proteins, consisting of Prc1 and Prc2, has central roles in epigenetic regulation of development, differentiation and maintenance of cell fate in embryonic stem cells [32,33]. Jarid2 recruits Prc2 to target genes and contributes to the establishment of high levels of lysine 27 methylation of Histone 3 (H3K27), leading to gene repression [34,35].

Notch signaling decides cell fates during development, and is critical for a variety of developmental programs, including in the central nervous system (reviewed by Yoon and Gaiano [36]). Four paralogues genes encoding the receptor proteins Notch 1–4 have been identified in vertebrates. Jarid2 has been shown to bind to the Notch1 locus, promoting di- and tri-methylation of lysine 9 on Histone 3 (H3K9me2 and H3K9me3), and repressing the transcription of Notch1 (Notch, Drosophila, homolog of, 1) [37]. Jarid2 knockout embryos have decreased levels of H3K9me2 and H3K9me3 and exhibit persistent high expression of Notch1. Although this mechanism was detected in the developing heart of the mice, Jarid2 might exert similar functions on Notch1 in other organ systems [37].

According to NCBI gene, Human Genome Variation Society (HGVS) and the 1000 Genome Project databases, no disease causing mutations have previously been described in *JARID2* in humans. However, haploinsufficiency of several histone methyltransferase genes is known to result in syndromic intellectual disability exemplified by: *MLL2* (myeloid/lymphoid or mixed-lineage leukemia 2) in Kabuki syndrome (MIM 147920) and *EHMT1* (Euchromatic Histone Methyltransferase 1) in Kleefstra syndrome (MIM 610253). Loss of function mutations in the lysine-specific demethylase *JARID1C*, another gene of the *JARID* family, also causes syndromic intellectual disability (MIM 300534). In addition, two studies have shown association between *JARID2* and ASDs, when using the data from the Autism Genetic Resource Exchange (AGRE) [38,39]. Based on these observations, we suggest that *JARID2* is likely to be the critical gene within SRO I, and the main gene causing the neurodevelopmental syndrome in Patients 1–4. In support of this, we performed an RNA expression study showing that the *JARID2* expression level was significantly decreased in these four patients (Figure 4A).

We found that our four patients with *JARID2* deletion had a similar facial appearance (Figure 1 and Table 1), in whom the most characteristic findings were prominent supraorbital ridges, deep set eyes, dark infraorbital circles and midface hypoplasia. These features were not consistently reported in the 10 previously described patients with *JARID2* deletion (Patients 1, 3, 6 from [1], A, C-G and J, Table 2 and Figure 3), but when we carefully inspected the photos available for seven of these individuals, a similar facial pattern could be confirmed. Although mild, the mentioned dysmorphic facial features were a consistent finding in these patients. Therefore, *JARID2* haploinsufficiency may result in a clinically recognizable neurodevelopmental syndrome.

In our four patients with *JARID2* haploinsufficiency, we also observed impairment of balance and coordination, and gait disturbance, which were more pronounced in Patients 1 and 4. The peculiar gait in Patients 1 and 4 (see Additional file 2: Video Patient 1 and Additional file 6: Video Patient 4) was interpreted as a sign of dyspraxia, suggesting a higher brain function deficit. Cerebral MRI examinations in these patients did not reveal pathological changes affecting the cerebellum. Patients 2 and 3 were not cooperative for sufficient assessment of putative dyspraxia. Impaired balance and coordination and/or gait disturbance were noted in four out of the 10 previously reported patients with *JARID2* deletion (Patients 6, A, C and J, Table 2), but a common etiology could not be identified. Developmental dyspraxia was described in Patient 6 [1], while clear indications of cerebellar ataxia were not reported in any of them. Muscular hypotonia was recorded in additional four patients without any specific reference to gait (Patients 1 from [1] and D-F).

SRO II, deleted in Patients 1–3 and 5, was defined by the 116 kb deletion in Patient 5 who presented with mild intellectual disability and behavioural abnormalities. This region included the genes *GMPRI* and *ATXN1*.

Guanosine monophosphate reductase (GMPR) catalyzes the irreversible deamination of guanosine monophosphate (GMP) to inosine monophosphate (IMP), and plays a role in maintaining the intracellular balance of A and G nucleotides [40]. According to NCBI gene, HGVS and the 1000 Genome Project databases, no disease causing mutations in this gene have previously been linked to neurodevelopmental syndromes.

*ATXN1* (ataxin-1), is well known for causing the dominantly inherited spinocerebellar ataxia type 1 (SCA1; OMIM 164400), mainly due to a gain of function mechanism upon expansion of a (CAG)n repeat. *Atxn1* knockout mice show deficits in spatial and learning memory, but not ataxic signs or neurodegeneration [41]. However, ablation of wild type *Atxn1* in the knock-in SCA1 mouse model (Atxn1^154Q/+^), resulted in a more severe SCA1 phenotype [42]. In addition, Atxn1 ^−/−^ mice shared transcriptional alterations with Atxn1^154Q/+^, possibly contributing to pathogenesis in SCA1 [35,43].

Similarly to *JARID2*, *ATXN1* also encodes for a transcriptional repressor protein, which acts on different pathways including Silencing Mediator of Retinoid and Tyroid Receptor (SMART), Histone Deacetylase (HDAC) 3 and 4, Capicua and LANP. In Drosophila and mouse cell lines, ATXN1 has been shown to act on the Notch pathway through interaction with the transcriptional corepressor CBF1 [44]. In addition, an association between SNPs in this gene and intelligence was reported in patients ascertained for ADHD [45].

Celestino–Soper *et al.* suggested that haploinsufficiency of *ATXN1* is associated with developmental delay and ASDs [1]. The statement relied on the evidence that 10 out of 13 previous patients with a deletion including this gene (Patients 1–3, 5 and 6 from [1], A, C-E, G, J-K and possibly B, which is uncertain due to low resolution mapping of the breakpoints) showed speech delay, ASDs, ADHD or other abnormal behaviour. Among these 10 patients, however, only one had a diagnosis of ASDs (Patient 1 from [1]), while another one possibly had ASDs but was not formally tested (Patient 2 from [1]), and a third one was defined as having a sensory processing brain disorder (Patient 6 from [1]). Furthermore, two patients (I and L), both had intact *ATXN1* and a diagnosis of ASDs, clearly indicating that ASDs in patients with 6p22-p24 deletion is not exclusively caused by *ATXN1* haploinsufficiency*.* Our four patients with *ATXN1* deletion exhibit behavioural abnormalities, but only Patient 3 fulfils the diagnostic criteria of ASDs. *ATXN1* haploinsufficiency therefore seems to result in intellectual disability with high risk of behavioural abnormalities, but not necessarily ASDs. We did not detect a significantly reduced *ATXN1* expression level in our four patients with a deletion of this gene, even though a trend of reduced levels was found (Figure 4B). The variation in expression levels between individuals, both for controls and patients, was seen for *ATXN1*. Because of this inter model individual variation, additional patients with an *ATXN1* deletion are needed to asses if this deletion leads to a reduced *ATXN1* expression. The RNA from the patients was isolated from leucocytes, and it cannot be excluded that a significantly reduced level would have been detected using RNA from specific brain regions.

We find it noteworthy that Patient 1, as the only one of our five patients, had variable length of the metacarpal and metatarsal bones of hands and feet. Although without X-ray images, a similar appearance of the foot, with broad and short halluxes and long 2^nd^ toes was described in Patient 1 from [1]. Haploinsufficiency of a gene located within these two patients' shared deletion overlap may cause disruption of skeletal growth.

In conclusion, chromosome 6p22-p24 haploinsufficiency results in a clinically heterogeneous neurodevelopmental syndrome, probably reflecting the different breakpoints and sizes and the lack of overlap between all deletions. The histone modifiers, *ATXN1* and *JARID2*, exert their effect on several target genes and possibly explain part of the phenotype. Interestingly, both ATXN1 and JARID2 act on the Notch pathway, an evolutionary conserved signalling pathway whose dysregulation can have a broad impact on developmental processes, including development of the central nervous system, which could be relevant in our patients.

We remark that *JARID2* deletion-patients in addition to cognitive impairment presented with gait disturbance and a characteristic facial appearance that may represent a clinically recognizable developmental syndrome, and that patients with an *ATXN1* deletion seemed to be characterized by intellectual disability and behavioural abnormalities. Simultaneous deletion of the two genes likely exacerbated the degree of intellectual disability in three of our patients. Previously both *JARID2* and *ATXN1* were linked to ASDs, but in our patients we detected a wider spectrum of behaviour abnormalities, rather than a well-defined diagnosis of ASDs, which only one of the patients had. Because *JARID2* haploinsufficiency appears to result in a clinically recognizable developmental syndrome, we suggest that this gene should be sequenced in future patients with a similar phenotype when a gene deletion is not present*.*

### Consent

This study was approved by the Regional Ethical Committee in Eastern Norway for research involving human subjects. Written consent for publication was obtained from the parents of all five patients.

## Competing interest

The authors declare no conflicts of interests.

## Authors’ contributions

Clinical evaluation of patients has been performed by ASP, AB, BT, MF and PS. OKR, AH, AS, TB and DM performed cytogenetic and molecular studies. TB drafted the manuscript. EF supervised and coordinated the study and critically revised the manuscript. DM, JRH, BT and PS critically revised the manuscript. All authors read and approved the final manuscript.

## Supplementary Material

Additional file 1 **Table S1.** RT-PCR primer sequences. Click here for file

Additional file 2 Video Patient 1 demonstrating impaired balance and coordination when asked to stand and jump on one leg and to walk in a straight line. Click here for file

Additional file 3 **Figure S1.** (A) X-ray images of hands of Patient 1 showing shortening of the 1st and 3rd-5th metacarpal bones left hand, and the 1st and 5th metacarpal bones right hand. (B) X-ray images of feet showing bilateral short 1st metatarsal bones. Click here for file

Additional file 4 Video Patient 2 demonstrating mild gait disturbance with broad-based legs. Click here for file

Additional file 5 **Video Patient 3 demonstrating stereotypic behavioural abnormalities with an intense compulsion for walking.** Note slightly broad-based legs. Click here for file

Additional file 6 Video Patient 4 demonstrating impaired balance and coordination when asked to stand and jump on one leg and to walk in a straight line. Click here for file
